# Supplementary material for: Plasma Polyunsaturated Fatty Acid Levels and Mental Health in Middle-Aged and Elderly Adults
Source: Nutrients. 2024 Nov 26;16(23):4065. doi: 10.3390/nu16234065 (PMC11643783; doi:10.3390/nu16234065)
Supplement: Supplementary file 1 [file nutrients-16-04065-s001.zip › nutrients-3293851-supplementary.pdf]

Supplementary Information

Plasma Polyunsaturated Fatty Acids Levels and Mental Health in Middle-Aged and Elderly Adults

Table S1. Definitions of mental health symptoms 2

Table S2. Definitions of brain white matter tracts 3

Table S3. Description of incidence rates of mental disorders by sex 4

Table S4. Description of plasma PUFA levels 5

Table S5. Associations between other PUFAs species and risks of mental disorders 6

Table S6. Associations of plasma total PUFAs with symptoms and stratified analysis by sex 7

Table S7. Associations of plasma omega-3 PUFAs with symptoms and stratified analysis by sex 8

Table S8. Associations of plasma DHA with symptoms and stratified analysis by sex 9

Table S9. Associations of plasma omega-6 PUFAs with symptoms and stratified analysis by sex 10

Table S10. Associations of plasma LA with symptoms and stratified analysis by sex 11

Table S11. Associations of plasma total PUFAs with the symptoms by age 12

Table S12. Associations of plasma omega-3 PUFAs with the symptoms by age 13

Table S13. Associations of plasma DHA with the symptoms by age 14

Table S14. Associations of plasma omega-6 PUFAs with the symptoms by age 15

Table S15. Associations of plasma LA with the symptoms by age 16

Table S16. Summary characteristics of MD and ISOVF values for white matter tracts 17

Table S17. Associations of plasma PUFAs with white matter tracts and stratified analysis by sex 18

Table S18. Associations of plasma PUFAs with white matter tracts by age 19

Table S19. Sensitivity analysis of the main associations 20

Table S1. Definitions of mental health symptoms

Item	UKB data field	Abbr	Field code
Subjective well-being	General happy	Happiness: general	20458
	Happiness with own health	Happiness: health	20459
	Belief that own life is meaningful	Meaningful: life	20460
PHQ-9	Recent thoughts of suicide or self-harm	Suicidal ideation	20513
	Trouble falling asleep, or sleeping too much	Sleeping problems	20517
	Recent changes in speed of moving or speaking	Psychomotor changes	20518
	Recent feelings of inadequacy	feelings of inadequacy	20507
	Recent feelings of tiredness or low energy	Fatigue	20519
	Recent feelings of depression	Depressed mood	20510
	Recent trouble concentrating on things	Cognitive problems	20508
	Recent poor appetite or overeating	Appetite changes	20511
	Recent lack of interest or pleasure in doing things	Anhedonia	20514
GAD-7	Recent inability to stop or control worrying	Worrying control	20509
	Recent restlessness	Restlessness	20516
	Recent trouble relaxing	Lack of relaxation	20515
	Recent easy annoyance or irritability	Irritability	20505
	Recent worrying too much about different things	Generalized worrying	20520
	Recent feelings of foreboding	Foreboding	20512
	Recent feelings or nervousness or anxiety	Anxiety feeling	20506

Table S2. Definitions of brain white matter tracts

White matter tract	Abbr	Unit	Filed code
**MD value**			
tract forceps major	FMA	AU	UKB data field 25525
tract forceps minor	FMI	AU	UKB data field 25526
tract middle cerebellar peduncle	MCP	AU	UKB data field 25531
tract acoustic radiation	AR	AU	UKB data field 25515 and 25516
tract anterior thalamic radiation	ATR	AU	UKB data field 25517 and 25518
tract cingulate gyrus part of cingulum	CGC	AU	UKB data field 25519 and 25520
tract corticospinal tract	CST	AU	UKB data field 25523 and 25524
tract inferior fronto-occipital fasciculus	IFOF	AU	UKB data field 25527 and 25528
tract inferior longitudinal fasciculus	ILF	AU	UKB data field 25529 and 25530
tract medial lemniscus	ML	AU	UKB data field 25532 and 25533
tract parahippocampal part of cingulum	CGH	AU	UKB data field 25521 and 25522
tract posterior thalamic radiation	PTR	AU	UKB data field 25534 and 25535
tract superior longitudinal fasciculus	SLF	AU	UKB data field 25536 and 25537
tract superior thalamic radiation	STR	AU	UKB data field 25538 and 25539
tract uncinate fasciculus	UF	AU	UKB data field 25540 and 25541
**ISOVF value**			
tract forceps major	FMA	AU	UKB data field 25714
tract forceps minor	FMI	AU	UKB data field 25715
tract middle cerebellar peduncle	MCP	AU	UKB data field 25720
tract acoustic radiation	AR	AU	UKB data field 25704 and 25705
tract anterior thalamic radiation	ATR	AU	UKB data field 25706 and 25707
tract cingulate gyrus part of cingulum	CGC	AU	UKB data field 25708 and 25709
tract corticospinal tract	CST	AU	UKB data field 25712 and 25713
tract inferior fronto-occipital fasciculus	IFOF	AU	UKB data field 25716 and 25717
tract inferior longitudinal fasciculus	ILF	AU	UKB data field 25718 and 25719
tract medial lemniscus	ML	AU	UKB data field 25721 and 25722
tract parahippocampal part of cingulum	CGH	AU	UKB data field 25710 and 25711
tract posterior thalamic radiation	PTR	AU	UKB data field 25723 and 25724
tract superior longitudinal fasciculus	SLF	AU	UKB data field 25725 and 25726
tract superior thalamic radiation	STR	AU	UKB data field 25727 and 25728
tract uncinate fasciculus	UF	AU	UKB data field 25729 and 25730

Table S3. Description of incidence rates of mental disorders by sex

Mental disorder	New events, No	Total person year, year	Incidence Rate, per 100,000 person-years
**All**			
Depressive disorder	3411	1361315	250.6
Anxiety disorder	3326	1363540	243.9
**Female**			
Depressive disorder	2100	728804	288.1
Anxiety disorder	2180	729385	298.9
**Male**			
Depressive disorder	1311	632511	207.3
Anxiety disorder	1146	634156	180.7

Table S4. Description of plasma PUFA levels

White matter tracts	P25	P50	P75	Mean	SD
Total PUFAs	4.431	4.929	5.468	4.981	0.797
Omega-3 PUFAs	0.375	0.494	0.642	0.528	0.219
DHA	0.180	0.223	0.277	0.235	0.083
Non-DHA omega-3 PUFAs	0.188	0.272	0.373	0.292	0.148
Omega-6 PUFAs	3.990	4.411	4.867	4.453	0.678
LA	2.953	3.372	3.828	3.418	0.682
Non-LA omega-6 PUFAs	0.936	1.026	1.125	1.036	0.148

Table S5. Associations between other PUFAs species and risks of mental disorders

Mental disorders[a]	Per Quartiles, mmol/L[d]				Per IQR
	Q1	Q2	Q3	Q4	
**Non-DHA omega-3 PUFAs[b]**					
Depressive disorder					
Model 1	Reference	0.91 (0.83, 1.00)	0.89 (0.81, 0.98)	0.85 (0.77, 0.93)	0.95 (0.91, 0.99)
Model 2	Reference	0.98 (0.88, 1.09)	0.92 (0.82, 1.03)	0.92 (0.82, 1.03)	0.97 (0.93, 1.02)
Model 3	Reference	0.98 (0.87, 1.10)	0.91 (0.81, 1.03)	0.90 (0.79, 1.01)	0.98 (0.93, 1.04)
Anxiety disorder					
Model 1	Reference	0.95 (0.86, 1.05)	0.91 (0.82, 1.00)	0.89 (0.80, 0.98)	0.96 (0.92, 1.00)
Model 2	Reference	0.96 (0.86, 1.07)	0.89 (0.80, 1.00)	0.88 (0.78, 0.98)	0.95 (0.90, 1.00)
Model 3	Reference	0.98 (0.87, 1.11)	0.89 (0.79, 1.01)	0.89 (0.79, 1.01)	0.96 (0.91, 1.01)
**Non-LA omega-6 PUFAs[c]**					
Depressive disorder					
Model 1	Reference	1.08 (0.97, 1.19)	1.12 (1.01, 1.24)	1.20 (1.09, 1.32)	1.10 (1.05, 1.15)
Model 2	Reference	1.08 (0.96, 1.21)	1.12 (1.00, 1.26)	1.18 (1.05, 1.32)	1.09 (1.04, 1.15)
Model 3	Reference	1.08 (0.95, 1.22)	1.13 (1.00, 1.28)	1.17 (1.03, 1.32)	1.09 (1.03, 1.15)
Anxiety disorder					
Model 1	Reference	1.07 (0.96, 1.18)	1.03 (0.93, 1.14)	1.08 (0.98, 1.19)	1.03 (0.99, 1.08)
Model 2	Reference	1.11 (0.99, 1.25)	1.06 (0.95, 1.19)	1.11 (0.99, 1.25)	1.03 (0.98, 1.09)
Model 3	Reference	1.12 (0.99, 1.27)	1.04 (0.92, 1.18)	1.09 (0.96, 1.24)	1.02 (0.96, 1.07)

Abbreviations: PUFAs, polyunsaturated fatty acids; DHA, docosahexaenoic acid; LA, linoleic acid. a Three model strategies was adopted for the analyses: Model 1 adjusted baseline characteristics including age, sex, WHR, and IMD; Model 2 additionally adjusted for healthy lifestyles (never drinking, healthy sleep pattern, healthy diet, and regular physical activity); Model 3 further additionally adjusted for systolic blood pressure, diastolic blood pressure, and blood glucose. b The plasma level of non-DHA omega-3 PUFAs was divided into quintiles: Q1 (<0.19 mmol/L), Q2 (0.19 to 0.27 mmol/L), Q3 (0.27 to 0.37 mmol/L), and Q4 (>0.37 mmol/L). c The plasma level of non-LA omega-6 PUFAs was divided into quartiles: Q1 (<0.94 mmol/L), Q2 (0.94 to 1.03 mmol/L), Q3 (1.03 to 1.13 mmol/L), and Q4 (>1.13 mmol/L).

Table S6. Associations of plasma total PUFAs with symptoms and stratified analysis by sex

Symptom	OR (95% CI)[a]	*P* value[b]	Sex		
			Male	Female	*P* for interaction[c]
**Subjective well-being**					
General happy	0.94 (0.76, 1.16)	0.720	0.90 (0.66, 1.22)	0.97 (0.72, 1.31)	0.827
Happiness with own health	0.77 (0.66, 0.89)	<0.001	0.78 (0.63, 0.96)	0.79 (0.64, 0.97)	0.721
Belief that own life is meaningful	0.79 (0.66, 0.95)	0.050	0.79 (0.60, 1.02)	0.81 (0.63, 1.05)	0.865
**PHQ-9**					
Recent thoughts of suicide or self-harm	0.82 (0.64, 1.05)	0.390	0.86 (0.60, 1.20)	0.80 (0.56, 1.14)	0.984
Trouble falling asleep, or sleeping too much	1.00 (0.91, 1.09)	0.980	1.08 (0.94, 1.23)	0.94 (0.83, 1.07)	0.187
Recent changes in speed of moving or speaking	0.88 (0.71, 1.10)	0.540	0.80 (0.57, 1.12)	0.93 (0.69, 1.26)	0.953
Recent feelings of inadequacy	0.97 (0.86, 1.09)	0.720	1.00 (0.84, 1.20)	0.96 (0.82, 1.12)	0.691
Recent feelings of tiredness or low energy	0.87 (0.79, 0.95)	0.010	0.91 (0.80, 1.04)	0.84 (0.74, 0.95)	0.378
Recent feelings of depression	0.97 (0.87, 1.09)	0.720	1.02 (0.86, 1.21)	0.94 (0.81, 1.10)	0.617
Recent trouble concentrating on things	0.96 (0.85, 1.09)	0.720	1.11 (0.94, 1.32)	0.85 (0.72, 1.01)	0.018
Recent poor appetite or overeating	0.80 (0.71, 0.91)	<0.001	0.97 (0.80, 1.18)	0.72 (0.62, 0.84)	0.022
Recent lack of interest or pleasure in doing things	0.92 (0.82, 1.04)	0.470	0.91 (0.76, 1.08)	0.95 (0.80, 1.12)	0.511
**GAD-7**					
Recent inability to stop or control worrying	0.97 (0.87, 1.08)	0.720	0.94 (0.79, 1.11)	0.99 (0.86, 1.14)	0.896
Recent restlessness	0.96 (0.83, 1.10)	0.720	0.83 (0.66, 1.04)	1.07 (0.88, 1.30)	0.174
Recent trouble relaxing	0.93 (0.84, 1.03)	0.390	1.01 (0.86, 1.17)	0.88 (0.77, 1.01)	0.236
Recent easy annoyance or irritability	0.93 (0.84, 1.03)	0.390	1.00 (0.86, 1.16)	0.87 (0.75, 1.00)	0.092
Recent worrying too much about different things	0.96 (0.87, 1.06)	0.720	0.93 (0.80, 1.08)	1.00 (0.88, 1.14)	0.650
Recent feelings of foreboding	1.01 (0.89, 1.14)	0.970	0.95 (0.78, 1.16)	1.04 (0.88, 1.22)	0.813
Recent feelings or nervousness or anxiety	1.02 (0.92, 1.13)	0.760	1.01 (0.86, 1.18)	1.04 (0.90, 1.19)	0.499

Abbreviations: OR, odds ratio. **a** Plasma PUFAs intake was divided into quartile, OR and corresponding 95% CI for quartile 1 was calculated with the highest quartiles as reference. **b** A low '*P* value FDR' (typically less than 0.05) suggests statistical significance. **c** A low '*P* for interaction ' (typically less than 0.05) suggests statistically significant.

Table S7. Associations of plasma omega-3 PUFAs with symptoms and stratified analysis by sex

Symptom	OR (95% CI)[a]	*P* value[b]	Sex		
			Male	Female	*P* for interaction[c]
**Subjective well-being**					
General happy	0.75 (0.61, 0.93)	0.020	0.58 (0.42, 0.79)	0.98 (0.73, 1.32)	0.010
Happiness with own health	0.72 (0.62, 0.83)	<0.001	0.75 (0.61, 0.93)	0.72 (0.58, 0.88)	0.734
Belief that own life is meaningful	0.67 (0.56, 0.80)	<0.001	0.60 (0.46, 0.78)	0.76 (0.59, 0.97)	0.212
**PHQ-9**					
Recent thoughts of suicide or self-harm	0.79 (0.62, 0.99)	0.050	0.79 (0.57, 1.09)	0.81 (0.58, 1.14)	0.798
Trouble falling asleep, or sleeping too much	0.90 (0.82, 0.99)	0.030	0.88 (0.77, 1.01)	0.92 (0.81, 1.04)	0.960
Recent changes in speed of moving or speaking	0.78 (0.63, 0.97)	0.030	0.76 (0.55, 1.03)	0.82 (0.62, 1.10)	0.367
Recent feelings of inadequacy	0.84 (0.75, 0.94)	0.010	0.87 (0.73, 1.04)	0.82 (0.70, 0.95)	0.996
Recent feelings of tiredness or low energy	0.80 (0.73, 0.88)	<0.001	0.80 (0.70, 0.91)	0.81 (0.72, 0.92)	0.828
Recent feelings of depression	0.83 (0.75, 0.93)	<0.001	0.77 (0.65, 0.91)	0.90 (0.78, 1.05)	0.082
Recent trouble concentrating on things	0.94 (0.83, 1.06)	0.320	0.97 (0.82, 1.15)	0.91 (0.77, 1.07)	0.649
Recent poor appetite or overeating	0.76 (0.67, 0.86)	<0.001	0.84 (0.69, 1.02)	0.73 (0.62, 0.85)	0.211
Recent lack of interest or pleasure in doing things	0.78 (0.70, 0.88)	<0.001	0.72 (0.61, 0.86)	0.85 (0.72, 1.00)	0.096
**GAD-7**					
Recent inability to stop or control worrying	0.88 (0.79, 0.98)	0.030	0.82 (0.69, 0.97)	0.93 (0.81, 1.07)	0.368
Recent restlessness	0.84 (0.73, 0.97)	0.030	0.69 (0.55, 0.87)	0.97 (0.80, 1.17)	0.026
Recent trouble relaxing	0.89 (0.80, 0.98)	0.030	0.81 (0.69, 0.94)	0.95 (0.83, 1.08)	0.204
Recent easy annoyance or irritability	0.87 (0.78, 0.96)	0.010	0.92 (0.79, 1.07)	0.82 (0.72, 0.94)	0.152
Recent worrying too much about different things	0.89 (0.80, 0.98)	0.030	0.80 (0.68, 0.93)	0.95 (0.84, 1.08)	0.049
Recent feelings of foreboding	0.96 (0.85, 1.08)	0.480	0.84 (0.69, 1.02)	1.04 (0.89, 1.22)	0.106
Recent feelings or nervousness or anxiety	0.92 (0.83, 1.01)	0.100	0.83 (0.71, 0.97)	0.99 (0.87, 1.13)	0.126

Abbreviations: OR, odds ratio. **a** Plasma omega-3 PUFAs intake was divided into quartile, OR and corresponding 95% CI for quartile 1 was calculated with the highest quartiles as reference. **b** A low '*P* value FDR' (typically less than 0.05) suggests statistical significance. **c** A low '*P* for interaction ' (typically less than 0.05) suggests statistically significant.

Table S8. Associations of plasma DHA with symptoms and stratified analysis by sex

Symptom	OR (95% CI)[a]	*P* value[b]	Sex		
			Male	Female	*P* for interaction[c]
**Subjective well-being**					
General happy	0.72 (0.58, 0.90)	0.010	0.63 (0.45, 0.86)	0.85 (0.63, 1.17)	0.043
Happiness with own health	0.67 (0.57, 0.78)	<0.001	0.68 (0.54, 0.85)	0.67 (0.55, 0.83)	0.605
Belief that own life is meaningful	0.74 (0.61, 0.89)	<0.001	0.78 (0.59, 1.01)	0.75 (0.58, 0.98)	0.854
**PHQ-9**					
Recent thoughts of suicide or self-harm	0.79 (0.62, 1.01)	0.080	0.85 (0.60, 1.19)	0.78 (0.55, 1.13)	0.771
Trouble falling asleep, or sleeping too much	0.86 (0.78, 0.94)	<0.001	0.85 (0.74, 0.97)	0.90 (0.79, 1.02)	0.374
Recent changes in speed of moving or speaking	0.74 (0.60, 0.92)	0.010	0.71 (0.51, 0.97)	0.88 (0.65, 1.21)	0.160
Recent feelings of inadequacy	0.84 (0.75, 0.95)	0.010	0.86 (0.72, 1.03)	0.85 (0.73, 1.00)	0.879
Recent feelings of tiredness or low energy	0.78 (0.71, 0.86)	<0.001	0.75 (0.66, 0.86)	0.83 (0.73, 0.95)	0.302
Recent feelings of depression	0.79 (0.71, 0.89)	<0.001	0.73 (0.62, 0.87)	0.87 (0.75, 1.02)	0.032
Recent trouble concentrating on things	0.91 (0.81, 1.03)	0.160	0.87 (0.73, 1.04)	0.94 (0.79, 1.12)	0.687
Recent poor appetite or overeating	0.66 (0.58, 0.74)	<0.001	0.61 (0.49, 0.76)	0.69 (0.59, 0.81)	0.962
Recent lack of interest or pleasure in doing things	0.73 (0.64, 0.82)	<0.001	0.68 (0.56, 0.81)	0.80 (0.67, 0.95)	0.074
**GAD-7**					
Recent inability to stop or control worrying	0.87 (0.78, 0.98)	0.030	0.84 (0.70, 1.00)	0.93 (0.80, 1.08)	0.181
Recent restlessness	0.76 (0.66, 0.88)	<0.001	0.66 (0.52, 0.82)	0.91 (0.75, 1.12)	0.010
Recent trouble relaxing	0.93 (0.84, 1.03)	0.180	0.84 (0.72, 0.99)	1.03 (0.89, 1.19)	0.109
Recent easy annoyance or irritability	0.84 (0.75, 0.93)	<0.001	0.84 (0.72, 0.99)	0.81 (0.70, 0.94)	0.540
Recent worrying too much about different things	0.94 (0.85, 1.04)	0.220	0.82 (0.70, 0.96)	1.05 (0.92, 1.21)	0.008
Recent feelings of foreboding	0.91 (0.80, 1.03)	0.160	0.83 (0.67, 1.01)	0.96 (0.82, 1.14)	0.309
Recent feelings or nervousness or anxiety	0.95 (0.85, 1.05)	0.300	0.90 (0.77, 1.05)	1.01 (0.88, 1.17)	0.101

Abbreviations: OR, odds ratio. **a** Plasma DHA intake was as divided into quartile, OR and corresponding 95% CI for quartile 1 was calculated with the highest quartiles as reference. **b** A low '*P* value FDR' (typically less than 0.05) suggests statistical significance. **c** A low '*P* for interaction ' (typically less than 0.05) suggests statistically significant.

Table S9. Associations of plasma omega-6 PUFAs with symptoms and stratified analysis by sex

Symptom	OR (95% CI)[a]	*P* value[b]	Sex		
			Male	Female	*P* for interaction[c]
**Subjective well-being**					
General happy	0.97 (0.79, 1.21)	0.900	1.01 (0.74, 1.36)	0.92 (0.68, 1.25)	0.711
Happiness with own health	0.78 (0.68, 0.91)	0.010	0.78 (0.63, 0.96)	0.79 (0.65, 0.97)	0.626
Belief that own life is meaningful	0.83 (0.70, 1.00)	0.230	0.81 (0.62, 1.05)	0.86 (0.67, 1.11)	0.986
**PHQ-9**					
Recent thoughts of suicide or self-harm	0.80 (0.62, 1.02)	0.260	0.83 (0.58, 1.18)	0.75 (0.53, 1.07)	0.926
Trouble falling asleep, or sleeping too much	0.99 (0.90, 1.08)	0.900	1.07 (0.93, 1.22)	0.93 (0.82, 1.05)	0.168
Recent changes in speed of moving or speaking	0.83 (0.67, 1.03)	0.260	0.81 (0.58, 1.11)	0.84 (0.63, 1.12)	0.929
Recent feelings of inadequacy	1.00 (0.89, 1.12)	0.990	1.04 (0.87, 1.24)	0.97 (0.83, 1.14)	0.539
Recent feelings of tiredness or low energy	0.85 (0.78, 0.93)	0.010	0.89 (0.78, 1.02)	0.82 (0.72, 0.93)	0.361
Recent feelings of depression	0.95 (0.85, 1.06)	0.620	1.00 (0.84, 1.18)	0.91 (0.78, 1.06)	0.344
Recent trouble concentrating on things	0.92 (0.82, 1.04)	0.390	1.10 (0.92, 1.30)	0.79 (0.67, 0.94)	0.008
Recent poor appetite or overeating	0.83 (0.73, 0.93)	0.010	1.00 (0.82, 1.21)	0.74 (0.63, 0.87)	0.023
Recent lack of interest or pleasure in doing things	0.95 (0.84, 1.07)	0.620	0.96 (0.80, 1.14)	0.93 (0.79, 1.10)	0.997
**GAD-7**					
Recent inability to stop or control worrying	0.97 (0.87, 1.08)	0.760	0.94 (0.79, 1.12)	0.99 (0.86, 1.14)	0.878
Recent restlessness	0.94 (0.82, 1.08)	0.630	0.87 (0.70, 1.08)	1.01 (0.83, 1.22)	0.273
Recent trouble relaxing	0.91 (0.83, 1.01)	0.260	0.99 (0.84, 1.15)	0.87 (0.76, 1.00)	0.125
Recent easy annoyance or irritability	0.92 (0.83, 1.02)	0.270	1.02 (0.88, 1.19)	0.85 (0.74, 0.98)	0.034
Recent worrying too much about different things	0.97 (0.88, 1.07)	0.760	0.95 (0.81, 1.10)	0.99 (0.87, 1.13)	0.977
Recent feelings of foreboding	0.99 (0.87, 1.12)	0.920	0.94 (0.77, 1.15)	1.01 (0.86, 1.18)	0.920
Recent feelings or nervousness or anxiety	1.02 (0.92, 1.13)	0.880	1.01 (0.86, 1.18)	1.03 (0.90, 1.18)	0.859

Abbreviations: OR, odds ratio. **a** Plasma omega-6 PUFAs intake as divided into quartile, OR and corresponding 95% CI for quartile 1 was calculated with the highest quartiles as reference. **b** A low '*P* value FDR' (typically less than 0.05) suggests statistical significance. **c** A low '*P* for interaction ' (typically less than 0.05) suggests statistically significant.

Table S10. Associations of plasma LA with symptoms and stratified analysis by sex

Symptom	OR (95% CI)[a]	*P* value[b]	Sex		
			Male	Female	*P* for interaction[c]
**Subjective well-being**					
General happy	1.01 (0.82, 1.25)	0.950	1.06 (0.79, 1.43)	0.94 (0.70, 1.28)	0.773
Happiness with own health	0.78 (0.68, 0.90)	0.020	0.77 (0.62, 0.95)	0.80 (0.65, 0.98)	0.574
Belief that own life is meaningful	0.88 (0.73, 1.05)	0.450	0.92 (0.72, 1.18)	0.85 (0.66, 1.09)	0.654
**PHQ-9**					
Recent thoughts of suicide or self-harm	0.90 (0.71, 1.14)	0.770	0.85 (0.60, 1.19)	0.93 (0.66, 1.32)	0.568
Trouble falling asleep, or sleeping too much	1.00 (0.91, 1.09)	0.950	1.05 (0.92, 1.20)	0.95 (0.84, 1.08)	0.311
Recent changes in speed of moving or speaking	0.93 (0.76, 1.15)	0.790	0.97 (0.71, 1.32)	0.88 (0.66, 1.16)	0.511
Recent feelings of inadequacy	1.06 (0.94, 1.19)	0.770	1.07 (0.90, 1.27)	1.06 (0.90, 1.23)	0.966
Recent feelings of tiredness or low energy	0.88 (0.80, 0.96)	0.030	0.94 (0.82, 1.06)	0.83 (0.73, 0.94)	0.308
Recent feelings of depression	0.97 (0.87, 1.09)	0.860	1.04 (0.89, 1.23)	0.92 (0.79, 1.07)	0.260
Recent trouble concentrating on things	0.98 (0.87, 1.10)	0.860	1.14 (0.96, 1.34)	0.86 (0.73, 1.02)	0.017
Recent poor appetite or overeating	0.86 (0.76, 0.97)	0.080	0.98 (0.80, 1.18)	0.80 (0.69, 0.94)	0.027
Recent lack of interest or pleasure in doing things	0.95 (0.84, 1.06)	0.770	0.98 (0.82, 1.16)	0.91 (0.77, 1.07)	0.626
**GAD-7**					
Recent inability to stop or control worrying	0.97 (0.87, 1.08)	0.860	0.95 (0.80, 1.12)	1.00 (0.87, 1.15)	0.910
Recent restlessness	0.94 (0.82, 1.08)	0.770	0.88 (0.71, 1.09)	0.99 (0.82, 1.19)	0.268
Recent trouble relaxing	0.90 (0.81, 0.99)	0.180	0.97 (0.83, 1.13)	0.85 (0.74, 0.97)	0.103
Recent easy annoyance or irritability	0.92 (0.83, 1.02)	0.400	1.01 (0.87, 1.17)	0.85 (0.74, 0.98)	0.036
Recent worrying too much about different things	0.97 (0.88, 1.07)	0.790	0.94 (0.81, 1.09)	0.99 (0.87, 1.13)	0.878
Recent feelings of foreboding	0.98 (0.87, 1.11)	0.860	0.99 (0.82, 1.21)	0.96 (0.82, 1.13)	0.738
Recent feelings or nervousness or anxiety	1.01 (0.91, 1.12)	0.950	1.00 (0.86, 1.17)	1.01 (0.89, 1.16)	0.939

Abbreviations: OR, odds ratio. **a** Plasma LA intake was as divided into quartile, OR and corresponding 95% CI for quartile 1 was calculated with the highest quartiles as reference. **b** A low '*P* value FDR' (typically less than 0.05) suggests statistical significance. **c** A low '*P* for interaction ' (typically less than 0.05) suggests statistically significant.

Table S11. Associations of plasma total PUFAs with the symptoms by age

Symptom	Age		
	<60	≥60	*P* for interaction[a]
**Subjective well-being**			
General happy	0.81 (0.63, 1.04)	1.43 (0.94, 2.21)	0.022
Happiness with own health	0.80 (0.67, 0.95)	0.71 (0.56, 0.91)	0.619
Belief that own life is meaningful	0.72 (0.58, 0.89)	1.02 (0.73, 1.42)	0.054
**PHQ-9**			
Recent thoughts of suicide or self-harm	0.74 (0.55, 0.99)	1.09 (0.68, 1.73)	0.144
Trouble falling asleep, or sleeping too much	0.96 (0.85, 1.07)	1.08 (0.93, 1.26)	0.117
Recent changes in speed of moving or speaking	0.87 (0.67, 1.13)	0.91 (0.62, 1.35)	0.933
Recent feelings of inadequacy	0.94 (0.82, 1.08)	1.04 (0.84, 1.29)	0.559
Recent feelings of tiredness or low energy	0.85 (0.76, 0.95)	0.89 (0.76, 1.03)	0.408
Recent feelings of depression	0.93 (0.82, 1.07)	1.05 (0.86, 1.29)	0.934
Recent trouble concentrating on things	0.90 (0.78, 1.04)	1.11 (0.89, 1.38)	0.175
Recent poor appetite or overeating	0.74 (0.64, 0.86)	0.97 (0.77, 1.23)	0.040
Recent lack of interest or pleasure in doing things	0.90 (0.78, 1.03)	0.97 (0.78, 1.20)	0.842
**GAD-7**			
Recent inability to stop or control worrying	0.94 (0.83, 1.08)	1.01 (0.83, 1.23)	0.777
Recent restlessness	0.94 (0.79, 1.12)	0.96 (0.74, 1.24)	0.845
Recent trouble relaxing	0.89 (0.79, 1.01)	1.01 (0.84, 1.21)	0.363
Recent easy annoyance or irritability	0.93 (0.82, 1.05)	0.91 (0.76, 1.09)	0.739
Recent worrying too much about different things	0.92 (0.82, 1.04)	1.06 (0.89, 1.25)	0.238
Recent feelings of foreboding	1.01 (0.87, 1.17)	0.98 (0.78, 1.23)	0.611
Recent feelings or nervousness or anxiety	0.99 (0.88, 1.12)	1.08 (0.91, 1.30)	0.787

Abbreviations: **a** A low '*P* for interaction ' (typically less than 0.05) suggests statistically significant.

Table S12. Associations of plasma omega-3 PUFAs with the symptoms by age

Symptom	Age		
	<60	≥60	*P* for interaction[a]
**Subjective well-being**			
General happy	0.69 (0.54, 0.88)	0.84 (0.56, 1.28)	0.297
Happiness with own health	0.71 (0.60, 0.85)	0.73 (0.57, 0.94)	0.769
Belief that own life is meaningful	0.58 (0.46, 0.72)	0.83 (0.61, 1.14)	0.007
**PHQ-9**			
Recent thoughts of suicide or self-harm	0.72 (0.54, 0.95)	0.92 (0.60, 1.44)	0.171
Trouble falling asleep, or sleeping too much	0.82 (0.74, 0.92)	1.09 (0.93, 1.27)	0.013
Recent changes in speed of moving or speaking	0.78 (0.60, 1.01)	0.75 (0.52, 1.09)	0.767
Recent feelings of inadequacy	0.82 (0.71, 0.94)	0.87 (0.70, 1.08)	0.374
Recent feelings of tiredness or low energy	0.73 (0.65, 0.82)	0.98 (0.84, 1.14)	0.005
Recent feelings of depression	0.81 (0.71, 0.93)	0.88 (0.72, 1.08)	0.637
Recent trouble concentrating on things	0.86 (0.75, 0.99)	1.15 (0.92, 1.44)	0.019
Recent poor appetite or overeating	0.73 (0.64, 0.85)	0.86 (0.68, 1.10)	0.092
Recent lack of interest or pleasure in doing things	0.74 (0.64, 0.86)	0.84 (0.68, 1.05)	0.523
**GAD-7**			
Recent inability to stop or control worrying	0.89 (0.78, 1.02)	0.83 (0.69, 1.01)	0.934
Recent restlessness	0.81 (0.68, 0.96)	0.89 (0.69, 1.15)	0.652
Recent trouble relaxing	0.87 (0.77, 0.98)	0.92 (0.76, 1.10)	0.583
Recent easy annoyance or irritability	0.88 (0.78, 0.99)	0.84 (0.70, 1.01)	0.756
Recent worrying too much about different things	0.89 (0.79, 1.00)	0.87 (0.73, 1.04)	0.982
Recent feelings of foreboding	0.97 (0.84, 1.12)	0.96 (0.77, 1.22)	0.560
Recent feelings or nervousness or anxiety	0.89 (0.79, 1.01)	0.96 (0.80, 1.15)	0.678

Abbreviations: **a** A low '*P* for interaction ' (typically less than 0.05) suggests statistically significant.

Table S13. Associations of plasma DHA with the symptoms by age

Symptom	Sex		
	<60	≥60	*P* for interaction[a]
**Subjective well-being**			
General happy	0.65 (0.50, 0.84)	0.98 (0.64, 1.53)	0.050
Happiness with own health	0.64 (0.53, 0.77)	0.75 (0.58, 0.96)	0.299
Belief that own life is meaningful	0.59 (0.47, 0.74)	1.16 (0.83, 1.62)	0.003
**PHQ-9**			
Recent thoughts of suicide or self-harm	0.70 (0.52, 0.93)	1.14 (0.71, 1.86)	0.047
Trouble falling asleep, or sleeping too much	0.80 (0.72, 0.90)	0.96 (0.82, 1.11)	0.085
Recent changes in speed of moving or speaking	0.69 (0.52, 0.89)	0.81 (0.56, 1.17)	0.382
Recent feelings of inadequacy	0.81 (0.70, 0.93)	0.94 (0.75, 1.17)	0.145
Recent feelings of tiredness or low energy	0.74 (0.66, 0.83)	0.88 (0.76, 1.02)	0.042
Recent feelings of depression	0.77 (0.67, 0.88)	0.85 (0.70, 1.05)	0.325
Recent trouble concentrating on things	0.82 (0.71, 0.95)	1.13 (0.91, 1.40)	0.009
Recent poor appetite or overeating	0.63 (0.54, 0.73)	0.76 (0.60, 0.97)	0.226
Recent lack of interest or pleasure in doing things	0.70 (0.61, 0.81)	0.78 (0.63, 0.97)	0.482
**GAD-7**			
Recent inability to stop or control worrying	0.85 (0.74, 0.97)	0.95 (0.78, 1.15)	0.406
Recent restlessness	0.75 (0.63, 0.90)	0.76 (0.59, 0.98)	0.920
Recent trouble relaxing	0.89 (0.79, 1.01)	1.02 (0.85, 1.23)	0.283
Recent easy annoyance or irritability	0.84 (0.74, 0.95)	0.83 (0.69, 0.99)	0.644
Recent worrying too much about different things	0.92 (0.82, 1.04)	0.98 (0.82, 1.17)	0.679
Recent feelings of foreboding	0.86 (0.74, 1.00)	1.05 (0.83, 1.32)	0.076
Recent feelings or nervousness or anxiety	0.93 (0.82, 1.05)	1.00 (0.83, 1.20)	0.638

Abbreviations: **a** A low '*P* for interaction ' (typically less than 0.05) suggests statistically significant.

Table S14. Associations of plasma omega-6 PUFAs with the symptoms by age

Symptom	Age		
	<60	≥60	*P* for interaction[a]
**Subjective well-being**			
General happy	0.82 (0.64, 1.05)	1.64 (1.07, 2.55)	0.006
Happiness with own health	0.78 (0.65, 0.93)	0.79 (0.62, 1.01)	0.881
Belief that own life is meaningful	0.76 (0.61, 0.95)	1.03 (0.74, 1.42)	0.074
**PHQ-9**			
Recent thoughts of suicide or self-harm	0.72 (0.54, 0.96)	1.02 (0.65, 1.61)	0.227
Trouble falling asleep, or sleeping too much	0.95 (0.85, 1.07)	1.05 (0.91, 1.22)	0.435
Recent changes in speed of moving or speaking	0.78 (0.60, 1.01)	0.96 (0.66, 1.41)	0.491
Recent feelings of inadequacy	0.96 (0.83, 1.10)	1.11 (0.89, 1.38)	0.484
Recent feelings of tiredness or low energy	0.84 (0.75, 0.94)	0.88 (0.76, 1.01)	0.610
Recent feelings of depression	0.92 (0.81, 1.05)	1.01 (0.82, 1.23)	0.954
Recent trouble concentrating on things	0.86 (0.74, 0.99)	1.08 (0.87, 1.34)	0.218
Recent poor appetite or overeating	0.76 (0.66, 0.88)	1.02 (0.81, 1.28)	0.047
Recent lack of interest or pleasure in doing things	0.93 (0.80, 1.07)	0.98 (0.79, 1.22)	0.976
**GAD-7**			
Recent inability to stop or control worrying	0.92 (0.80, 1.05)	1.08 (0.89, 1.30)	0.234
Recent restlessness	0.94 (0.79, 1.11)	0.94 (0.73, 1.20)	0.899
Recent trouble relaxing	0.87 (0.77, 0.99)	1.01 (0.84, 1.21)	0.230
Recent easy annoyance or irritability	0.92 (0.81, 1.04)	0.92 (0.77, 1.10)	0.949
Recent worrying too much about different things	0.91 (0.81, 1.02)	1.11 (0.94, 1.32)	0.056
Recent feelings of foreboding	0.98 (0.84, 1.13)	1.01 (0.81, 1.27)	0.810
Recent feelings or nervousness or anxiety	0.98 (0.87, 1.11)	1.11 (0.93, 1.32)	0.462

Abbreviations: **a** A low '*P* for interaction ' (typically less than 0.05) suggests statistically significant.

Table S15. Associations of plasma LA with the symptoms by age

Symptom	Age		
	<60	≥60	*P* for Interaction[a]
**Subjective well-being**			
General happy	0.88 (0.69, 1.12)	1.58 (1.02, 2.46)	0.026
Happiness with own health	0.78 (0.66, 0.94)	0.78 (0.61, 0.99)	0.430
Belief that own life is meaningful	0.79 (0.64, 0.98)	1.12 (0.80, 1.55)	0.106
**PHQ-9**			
Recent thoughts of suicide or self-harm	0.82 (0.62, 1.10)	1.11 (0.71, 1.75)	0.286
Trouble falling asleep, or sleeping too much	0.95 (0.85, 1.07)	1.07 (0.93, 1.24)	0.385
Recent changes in speed of moving or speaking	0.87 (0.68, 1.12)	1.07 (0.75, 1.54)	0.720
Recent feelings of inadequacy	1.02 (0.89, 1.17)	1.14 (0.92, 1.41)	0.594
Recent feelings of tiredness or low energy	0.86 (0.77, 0.96)	0.91 (0.78, 1.05)	0.785
Recent feelings of depression	0.95 (0.83, 1.09)	1.02 (0.84, 1.24)	0.866
Recent trouble concentrating on things	0.91 (0.79, 1.05)	1.14 (0.92, 1.41)	0.296
Recent poor appetite or overeating	0.80 (0.69, 0.92)	1.03 (0.82, 1.28)	0.038
Recent lack of interest or pleasure in doing things	0.92 (0.80, 1.06)	0.99 (0.81, 1.22)	0.874
**GAD-7**			
Recent inability to stop or control worrying	0.96 (0.84, 1.09)	0.99 (0.82, 1.20)	0.671
Recent restlessness	0.95 (0.80, 1.12)	0.92 (0.73, 1.17)	0.850
Recent trouble relaxing	0.85 (0.76, 0.96)	1.00 (0.84, 1.19)	0.218
Recent easy annoyance or irritability	0.89 (0.79, 1.01)	0.97 (0.82, 1.16)	0.736
Recent worrying too much about different things	0.92 (0.82, 1.04)	1.06 (0.89, 1.25)	0.152
Recent feelings of foreboding	0.99 (0.86, 1.15)	0.94 (0.76, 1.17)	0.869
Recent feelings or nervousness or anxiety	0.98 (0.87, 1.11)	1.06 (0.90, 1.27)	0.561

Abbreviations: **a** A low '*P* for interaction ' (typically less than 0.05) suggests statistically significant.

Table S16. Summary characteristics of MD and ISOVF values for white matter tracts

White matter tracts[a]	P25[b]	P50	P75	Mean	SD
**MD value**					
FMA	0.0008636	0.0008960	0.0009336	0.0009016	0.0000545
FMI	0.0008137	0.0008348	0.0008578	0.0008364	0.0000348
MCP	0.0007093	0.0007519	0.0007954	0.0007562	0.0000667
AR	0.0007657	0.0007855	0.0008072	0.0007873	0.0000334
ATR	0.0007635	0.0007837	0.0008087	0.0007892	0.0000395
CGC	0.0007497	0.0007655	0.0007831	0.0007666	0.0000294
CST	0.0007620	0.0007771	0.0007933	0.0007780	0.0000259
IFOF	0.0007886	0.0008082	0.0008303	0.0008112	0.0000332
ILF	0.0007964	0.0008160	0.0008385	0.0008191	0.0000336
ML	0.0008644	0.0008898	0.0009155	0.0008901	0.0000399
CGH	0.0008532	0.0008823	0.0009185	0.0008927	0.0000608
PTR	0.0008104	0.0008331	0.0008607	0.0008397	0.0000454
SLF	0.0007279	0.0007462	0.0007668	0.0007498	0.0000322
STR	0.0007437	0.0007596	0.0007779	0.0007620	0.0000303
UF	0.0007820	0.0008031	0.0008257	0.0008051	0.0000349
**ISOVF value**					
FMA	0.1280918	0.1449935	0.1651005	0.1482482	0.0280416
FMI	0.0792047	0.0885382	0.0985876	0.0894943	0.0154535
MCP	0.1038500	0.1282965	0.1539165	0.1313557	0.0377100
AR	0.0591979	0.0691912	0.0804112	0.0706767	0.0168418
ATR	0.0518810	0.0601817	0.0702904	0.0623923	0.0151436
CGC	0.0394836	0.0485125	0.0586866	0.0499390	0.0152801
CST	0.1122621	0.1213275	0.1308278	0.1219329	0.0144617
IFOF	0.0551463	0.0624696	0.0709582	0.0637699	0.0127270
ILF	0.0592502	0.0672470	0.0757232	0.0683171	0.0134312
ML	0.1406336	0.1558532	0.1718765	0.1568186	0.0244699
CGH	0.0632184	0.0792232	0.1019974	0.0874901	0.0370535
PTR	0.0702231	0.0808733	0.0938549	0.0842034	0.0210825
SLF	0.0711076	0.0790639	0.0885008	0.0807287	0.0143890
STR	0.0729523	0.0806165	0.0891614	0.0815915	0.0136257
UF	0.0267439	0.0326409	0.0402310	0.0348628	0.0135310

Abbreviations: SD, standard deviation. **a** The abbreviations of the brain imaging phenotypes were shown in Table S2.

Table S17. Associations of plasma PUFAs with white matter tracts and stratified analysis by sex

Phenotypes	*β* (95% CI)[a]	*P* value	*P* value FDR[b]	Sex		*P* for interaction[c]
				Male	Female	
**MD value**						
FMA	-0.079 (-0.150, -0.008)	0.028	0.048	-0.100 (-0.201, 0.000)	-0.049 (-0.150, 0.052)	0.490
FMI	-0.109 (-0.177, -0.041)	0.002	0.010	-0.102 (-0.198, -0.006)	-0.120 (-0.217, -0.023)	0.753
MCP	0.012 (-0.057, 0.082)	0.727	0.727	0.039 (-0.060, 0.138)	0.003 (-0.097, 0.102)	0.511
AR	-0.095 (-0.165, -0.024)	0.008	0.024	-0.088 (-0.188, 0.011)	-0.102 (-0.202, -0.002)	0.607
ATR	-0.063 (-0.125, -0.001)	0.046	0.063	-0.077 (-0.166, 0.011)	-0.040 (-0.129, 0.048)	0.820
CGC	-0.050 (-0.117, 0.017)	0.141	0.176	-0.067 (-0.161, 0.028)	-0.037 (-0.132, 0.058)	0.900
CST	-0.141 (-0.210, -0.073)	<0.001	<0.001	-0.107 (-0.204, -0.010)	-0.180 (-0.277, -0.082)	0.043
IFOF	-0.107 (-0.175, -0.039)	0.002	0.010	-0.161 (-0.258, -0.065)	-0.044 (-0.141, 0.053)	0.172
ILF	-0.105 (-0.173, -0.036)	0.003	0.011	-0.165 (-0.262, -0.068)	-0.037 (-0.135, 0.060)	0.205
ML	-0.017 (-0.090, 0.055)	0.637	0.727	-0.039 (-0.142, 0.064)	-0.006 (-0.109, 0.098)	0.701
CGH	-0.015 (-0.086, 0.056)	0.679	0.727	0.009 (-0.092, 0.110)	-0.032 (-0.133, 0.069)	0.309
PTR	-0.074 (-0.140, -0.007)	0.030	0.048	-0.069 (-0.163, 0.026)	-0.073 (-0.168, 0.022)	0.738
SLF	-0.089 (-0.158, -0.021)	0.011	0.027	-0.137 (-0.234, -0.040)	-0.038 (-0.136, 0.060)	0.255
STR	-0.072 (-0.137, -0.006)	0.032	0.048	-0.078 (-0.170, 0.015)	-0.066 (-0.159, 0.028)	0.731
UF	-0.076 (-0.144, -0.007)	0.030	0.048	-0.119 (-0.216, -0.022)	-0.018 (-0.116, 0.079)	0.415
**ISOVF value**						
FMA	-0.049 (-0.122, 0.023)	0.181	0.348	-0.074 (-0.177, 0.029)	-0.014 (-0.118, 0.089)	0.524
FMI	-0.084 (-0.157, -0.011)	0.024	0.120	-0.103 (-0.207, 0.000)	-0.075 (-0.179, 0.029)	0.848
MCP	0.001 (-0.069, 0.071)	0.979	0.979	0.056 (-0.042, 0.155)	-0.038 (-0.137, 0.061)	0.171
AR	-0.060 (-0.132, 0.012)	0.104	0.260	-0.064 (-0.166, 0.038)	-0.062 (-0.165, 0.040)	0.908
ATR	-0.032 (-0.102, 0.037)	0.362	0.494	-0.074 (-0.172, 0.025)	0.009 (-0.090, 0.108)	0.382
CGC	-0.011 (-0.083, 0.061)	0.765	0.820	-0.064 (-0.166, 0.039)	0.032 (-0.071, 0.134)	0.215
CST	-0.124 (-0.196, -0.052)	0.001	0.015	-0.107 (-0.209, -0.004)	-0.141 (-0.244, -0.038)	0.258
IFOF	-0.084 (-0.156, -0.012)	0.022	0.120	-0.176 (-0.278, -0.074)	0.012 (-0.091, 0.115)	0.029
ILF	-0.073 (-0.144, -0.002)	0.044	0.165	-0.174 (-0.274, -0.074)	0.027 (-0.074, 0.128)	0.020
ML	-0.017 (-0.089, 0.056)	0.649	0.749	-0.028 (-0.131, 0.075)	-0.017 (-0.120, 0.087)	0.781
CGH	0.040 (-0.030, 0.111)	0.263	0.394	0.064 (-0.036, 0.164)	0.022 (-0.079, 0.122)	0.492
PTR	-0.045 (-0.114, 0.025)	0.209	0.348	-0.066 (-0.165, 0.033)	-0.024 (-0.123, 0.076)	0.934
SLF	-0.062 (-0.132, 0.009)	0.088	0.260	-0.156 (-0.257, -0.056)	0.033 (-0.068, 0.134)	0.012
STR	-0.047 (-0.117, 0.023)	0.187	0.348	-0.079 (-0.178, 0.021)	-0.022 (-0.122, 0.078)	0.567
UF	-0.025 (-0.095, 0.045)	0.482	0.603	-0.072 (-0.172, 0.027)	0.031 (-0.069, 0.131)	0.269

Abbreviations: FDR, false discovery rate. a Plasma PUFA level was divided into quartiles, *β* value and corresponding (95% CI) was calculated with highest quartile as reference. b A low 'P value FDR' (typically less than 0.05) suggests statistical significance. c A low 'P for interaction ' (typically less than 0.05) suggests statistically significant.

Table S18. Associations of plasma PUFAs with white matter tracts by age

Phenotypes[a]	Age		*P* for interaction
	<60	≥60	
**MD value**			
FMA	-0.072 (-0.158, 0.014)	-0.110 (-0.234, 0.014)	0.397
FMI	-0.056 (-0.138, 0.027)	-0.227 (-0.345, -0.108)	0.038
MCP	0.056 (-0.028, 0.141)	-0.088 (-0.210, 0.034)	0.064
AR	-0.095 (-0.181, -0.010)	-0.099 (-0.221, 0.024)	0.769
ATR	0.008 (-0.067, 0.083)	-0.221 (-0.330, -0.113)	0.001
CGC	-0.040 (-0.121, 0.041)	-0.078 (-0.194, 0.038)	0.564
CST	-0.134 (-0.217, -0.052)	-0.162 (-0.281, -0.042)	0.456
IFOF	-0.058 (-0.140, 0.025)	-0.223 (-0.342, -0.105)	0.019
ILF	-0.058 (-0.141, 0.025)	-0.211 (-0.331, -0.091)	0.026
ML	-0.016 (-0.104, 0.072)	-0.021 (-0.147, 0.106)	0.984
CGH	0.015 (-0.071, 0.101)	-0.087 (-0.211, 0.037)	0.184
PTR	0.009 (-0.072, 0.089)	-0.252 (-0.368, -0.135)	0.001
SLF	-0.040 (-0.123, 0.044)	-0.207 (-0.327, -0.087)	0.018
STR	-0.010 (-0.090, 0.069)	-0.207 (-0.321, -0.092)	0.005
UF	-0.023 (-0.106, 0.060)	-0.205 (-0.324, -0.086)	0.018
**ISOVF value**			
FMA	-0.069 (-0.156, 0.019)	-0.019 (-0.146, 0.107)	0.927
FMI	-0.067 (-0.155, 0.021)	-0.113 (-0.241, 0.014)	0.629
MCP	0.044 (-0.040, 0.128)	-0.098 (-0.219, 0.023)	0.072
AR	-0.083 (-0.171, 0.004)	-0.012 (-0.138, 0.113)	0.511
ATR	0.000 (-0.084, 0.084)	-0.104 (-0.225, 0.017)	0.103
CGC	-0.032 (-0.119, 0.056)	0.033 (-0.093, 0.158)	0.690
CST	-0.105 (-0.193, -0.018)	-0.174 (-0.300, -0.047)	0.152
IFOF	-0.066 (-0.153, 0.022)	-0.136 (-0.262, -0.010)	0.187
ILF	-0.045 (-0.131, 0.041)	-0.143 (-0.267, -0.019)	0.110
ML	0.007 (-0.081, 0.095)	-0.067 (-0.193, 0.060)	0.420
CGH	0.078 (-0.007, 0.164)	-0.049 (-0.172, 0.074)	0.110
PTR	0.013 (-0.071, 0.098)	-0.171 (-0.292, -0.049)	0.034
SLF	-0.024 (-0.110, 0.061)	-0.151 (-0.275, -0.028)	0.037
STR	-0.017 (-0.102, 0.068)	-0.120 (-0.242, 0.003)	0.145
UF	0.018 (-0.067, 0.103)	-0.130 (-0.252, -0.007)	0.060

Abbreviations: **a** The abbreviations of the phenotypes were shown in Table S2. **b** A low '*P* for interaction' (typically less than 0.05) suggests statistically significant.

Table S19. Sensitivity analysis of the main associations

Disorder	Sensitivity analysis[a]	Q1	Q4	*P* value
**Total PUFAs**				
Depressive disorder	Sensitivity analysis 1	Reference	0.80 (0.70, 0.90)	<0.001
	Sensitivity analysis 2	Reference	0.80 (0.71, 0.91)	0.001
	Sensitivity analysis 3	Reference	0.79 (0.69, 0.90)	<0.001
	Sensitivity analysis 4	Reference	0.81 (0.72, 0.92)	0.001
	Sensitivity analysis 5	Reference	0.85 (0.72, 1.01)	0.065
Anxiety disorder	Sensitivity analysis 1	Reference	0.82 (0.72, 0.93)	0.002
	Sensitivity analysis 2	Reference	0.82 (0.72, 0.93)	0.002
	Sensitivity analysis 3	Reference	0.85 (0.74, 0.98)	0.028
	Sensitivity analysis 4	Reference	0.84 (0.74, 0.95)	0.006
	Sensitivity analysis 5	Reference	0.88 (0.74, 1.05)	0.168
**Omega-3 PUFAs**				
Depressive disorder	Sensitivity analysis 1	Reference	0.85 (0.75, 0.96)	0.012
	Sensitivity analysis 2	Reference	0.88 (0.77, 1.00)	0.047
	Sensitivity analysis 3	Reference	0.87 (0.76, 0.99)	0.039
	Sensitivity analysis 4	Reference	0.88 (0.78, 1.00)	0.046
	Sensitivity analysis 5	Reference	0.89 (0.77, 1.01)	0.078
Anxiety disorder	Sensitivity analysis 1	Reference	0.82 (0.72, 0.93)	0.002
	Sensitivity analysis 2	Reference	0.83 (0.73, 0.95)	0.005
	Sensitivity analysis 3	Reference	0.89 (0.77, 1.03)	0.107
	Sensitivity analysis 4	Reference	0.84 (0.75, 0.95)	0.007
	Sensitivity analysis 5	Reference	0.85 (0.74, 0.97)	0.018
**DHA**				
Depressive disorder	Sensitivity analysis 1	Reference	0.79 (0.69, 0.90)	<0.001
	Sensitivity analysis 2	Reference	0.81 (0.71, 0.92)	0.002
	Sensitivity analysis 3	Reference	0.79 (0.69, 0.91)	0.001
	Sensitivity analysis 4	Reference	0.82 (0.72, 0.94)	0.003
	Sensitivity analysis 5	Reference	0.88 (0.77, 1.01)	0.065
Anxiety disorder	Sensitivity analysis 1	Reference	0.78 (0.69, 0.89)	<0.001
	Sensitivity analysis 2	Reference	0.80 (0.70, 0.91)	0.001
	Sensitivity analysis 3	Reference	0.81 (0.70, 0.94)	0.005
	Sensitivity analysis 4	Reference	0.82 (0.72, 0.93)	0.002
	Sensitivity analysis 5	Reference	0.86 (0.75, 0.98)	0.025
**Omega-6 PUFAs**				
Depressive disorder	Sensitivity analysis 1	Reference	0.79 (0.70, 0.90)	<0.001
	Sensitivity analysis 2	Reference	0.79 (0.70, 0.89)	<0.001
	Sensitivity analysis 3	Reference	0.81 (0.71, 0.93)	0.002
	Sensitivity analysis 4	Reference	0.80 (0.71, 0.90)	<0.001
	Sensitivity analysis 5	Reference	0.86 (0.72, 1.02)	0.079

	Sensitivity analysis 1	Reference	0.84 (0.74, 0.95)	0.006
	Sensitivity analysis 2	Reference	0.84 (0.74, 0.95)	0.006
Anxiety disorder	Sensitivity analysis 3	Reference	0.89 (0.77, 1.02)	0.096
	Sensitivity analysis 4	Reference	0.85 (0.75, 0.96)	0.011
	Sensitivity analysis 5	Reference	0.92 (0.77, 1.10)	0.369
**LA**				
	Sensitivity analysis 1	Reference	0.78 (0.69, 0.89)	<0.001
	Sensitivity analysis 2	Reference	0.77 (0.68, 0.88)	<0.001
Depressive disorder	Sensitivity analysis 3	Reference	0.78 (0.68, 0.89)	<0.001
	Sensitivity analysis 4	Reference	0.77 (0.69, 0.87)	<0.001
	Sensitivity analysis 5	Reference	0.83 (0.70, 0.98)	0.027
	Sensitivity analysis 1	Reference	0.84 (0.74, 0.96)	0.009
	Sensitivity analysis 2	Reference	0.83 (0.73, 0.94)	0.004
Anxiety disorder	Sensitivity analysis 3	Reference	0.89 (0.77, 1.03)	0.110
	Sensitivity analysis 4	Reference	0.84 (0.75, 0.95)	0.007
	Sensitivity analysis 5	Reference	0.92 (0.78, 1.10)	0.367

a Sensitivity analysis 1: restricting participants to white European ancestry; sensitivity analysis 2: excluding participants who had been diagnosed with a targeted disease event within the first two years of follow-up; sensitivity analysis 3: advancing the deadline for follow-up to December 31, 2019; sensitivity analysis 4: additionally adjusting for no heavy alcohol intake in the model 3; sensitivity 5: additionally adjusting the plasma cholesterol and triglyceride in the model 3.
